# The Measurement of Intraocular Biomarkers in Various Stages of Proliferative Diabetic Retinopathy Using Multiplex xMAP Technology

**DOI:** 10.1155/2015/424783

**Published:** 2015-09-27

**Authors:** Stepan Rusnak, Jindra Vrzalova, Marketa Sobotova, Lenka Hecova, Renata Ricarova, Ondrej Topolcan

**Affiliations:** ^1^Department of Ophthalmology, University Hospital Pilsen, Alej Svobody 80, 304 60 Plzen, Czech Republic; ^2^Department of Nuclear Medicine, Laboratory of Immunoanalysis, University Hospital Pilsen, Dr. E. Benese 13, 305 99 Plzen, Czech Republic; ^3^Central Radioisotopic Laboratory, Faculty of Medicine in Pilsen, Charles University in Prague, Dr. E. Benese 13, 305 99 Plzen, Czech Republic

## Abstract

*Purpose*. To determine the intraocular levels of growth factors and cytokines in patients with various degrees of severity of proliferative diabetic retinopathy (PDR) using multiplex xMAP technology.* Methods*. A prospective cohort study of 61 eyes from 56 patients who were divided into 3 groups based on the severity of PDR. Patients in group number 1 are those who presented PDR with no need of repeated surgical intervention; patients in group number 2 had repeated vitreous bleeding; and patients in group number 3 had refractory neovascular glaucoma. The concentrations of proangiogenic, antiangiogenic, inflammatory, and neurotrophic factors were measured in intraocular fluid. The results were also compared with levels of factors measured in 50 eyes from 50 patients prior to senile cataract surgery (control group).* Results*. Patients with refractory neovascular glaucoma (the highest clinical severity group) had higher levels of interleukin 6 (IL-6) (median1 37.19; median3 384.74; *P* = .00096), transforming growth factor beta 1 (TGF*β*-1) (median1 49.00; median3 414.40; *P* = .0017), and vascular endothelial growth factor (VEGF) (median1 211.62; median3 352.82; *P* = .0454) compared with other PDR patients.* Conclusions*. Results of our study imply that levels of IL-6, TGF*β*-1, and VEGF correlate with the severity of PDR.

## 1. Introduction

Diabetes mellitus is one of the most common endocrine disorders in the world; it affected roughly 6% of the global population ca. in the year 2000, and it is estimated that it will affect 300 million people in 2025 [1]. Diabetic retinopathy affects 35% of the patients in the diabetic population [2] and is the main cause of permanent vision loss in the working population [3]. Proliferative diabetic retinopathy (PDR) is characterized by the pathological formation of retinal blood vessels. Despite progress in diagnostics and therapies, PDR leads to a terminal stage of therapeutically unmanageable neovascularization that is characterized by the development of secondary neovascular glaucoma in a number of cases.

Retinal hypoxia is a major driving force for retinal neovascularization that increases hypoxia inducible factor (HIF) levels and launches a cascade of the production of cytokines and growth factors. Since the discovery of the proangiogenic role of vascular endothelial growth factor (VEGF) in PDR, changes in the levels of a number of other proangiogenic factors, such as those in the insulin-like growth factor family (IGF), hepatocyte growth factor (HGF), basic fibroblast growth factor (b-FGF), platelet-derived growth factor (PDGF), proinflammatory cytokines, and angiopoietin, have been demonstrated. However, the intraocular synthesis of angiogenic factors is counterbalanced by the synthesis of antiangiogenic factors, including *γ*-interferon inducible protein 10 (IP-10), the pigment epithelium-derived factor (PEDF), transforming growth factor beta (TGF*β*), thrombospondin (TSP), endostatin, angiostatin, and somatostatin [4, 5]. Fluorescein angiography, or more recently ultra wide-field fluorescein angiography, is used to determine the scope of neovascularization or ischemia [6]. This method is an image-processing technique for angiographic mapping of the retina. It enables the most recent stage of retinal angiogenesis to be described, but it is not an objective risk assessment technique. The measurement of intraocular biomarkers is emerging as a novel possibility for patient stratification. Because neovascularization results from an imbalance in proangiogenic and antiangiogenic factors, a multiplex analytical tool for monitoring the levels of several factors in a small sample volume is necessary to describe this process. A combination of immunoanalysis and flow cytometry [7], called xMAP technology, is one of the most promising multiplex technologies in clinical research to date.

In our study, the concentration levels of epidermal growth factor (EGF), interleukin 6 (IL-6), VEGF, tumor necrosis factor alfa (TNF-*α*), interleukin 8 (IL-8), IP-10, monocyte chemoattractant protein 1 (MCP-1), PDGF, TGF*β*-1, fractalkine, interleukin 10 (IL-10), interferon gamma (IFN-*γ*), fibroblast growth factor 2 (FGF-2), brain-derived neurotrophic factor (BDNF), ciliary neurotrophic factor (CNTF), and RANTES in samples of the aqueous humour from a group of PDR patients were measured using xMAP technology. The PDR cohort was further divided into three subgroups on the basis of clinical severity and these subgroups were compared with a control group. Our aim was to demonstrate that a biomarker panel measurement using multiplex immunoanalysis is applicable as a diagnostic and prognostic method in ophthalmology.

## 2. Materials and Methods

### 2.1. Patient Cohort

Patients undergoing treatment for PDR at the University Hospital in Pilsen during 2008–2010 were enrolled in this institutional prospective cohort study.

The patients with PDR were divided into groups according to the severity of their pathologies. Group 1 included 41 eyes from 37 patients with PDR who had no need for repeated surgical intervention (for better understanding, this group consists of 26 patients with PDR and vitreous bleeding, 5 patients with PDR and tractional retinal detachment, 5 patients with PDR, vitreous bleeding, and tractional retinal detachment, 4 patients with PDR and exudative maculopathy, and 1 patient with PDR with fibroproliferation), group 2 was composed of 11 eyes of nine patients who had repeated vitreous bleeding, and group 3 included 10 eyes from 10 patients with refractory neovascular glaucoma, which represents the most severe stage of the disease. The control group (group 0) was composed of 50 eyes from 50 preoperative senile cataract patients. Small samples (approximately 50 *μ*L) of intraocular fluid from the aqueous humour of each participant were obtained under topical anesthesia from the anterior chamber of each eye by means of aspiration using a fine 30-gauge needle that was attached to a syringe.

### 2.2. Multiplex Analysis

All specimens were frozen immediately. Samples were stored at −80°C until they were analyzed. No more than one freeze-thaw cycle was allowed prior to analysis. The protein concentrations in the aqueous humour were measured using multiplex xMAP technology on a Luminex 100 instrument with commercially available panels from Millipore Corporation (Billerica, MA, USA), MILLIPLEX MAP Human Cytokine/Chemokine Panel, and MILLIPLEX MAP TGF*β*-1. The procedures were performed according to the manufacturer's instructions, and the control samples that were provided within the kits were assayed in each analysis. The xMAP technology that was applied is a combination of immunoanalysis and flow cytometry based on bead particles that can be distinguished by internal dyes, as described, for example, by Kellar and Iannone [7]. In our study, the levels of EGF, IL-6, VEGF, TNF-*α*, IL-8, IP-10, MCP-1, PDGF AA, TGF*β*-1, fractalkine, PDGF AB/BB, IL-10, IFN-*γ*, FGF-2, CNTF, BDNF, and RANTES were studied.

### 2.3. Statistical Methods

A descriptive statistic was calculated for each of the markers. The results under the calibration curve ranges were stated as the value of the lowest calibration point. The Mann-Whitney* U* test (independent samples) and Kruskal-Wallis test were used to compare marker levels between groups. Borderline significance was determined to be reflected by *P* values ranging from 0.05 to 0.0001, and significance was reflected by *P* values below 0.0001. MedCalc 11.2 statistics software was used for analysis.

## 3. Results

**Figure 1:**
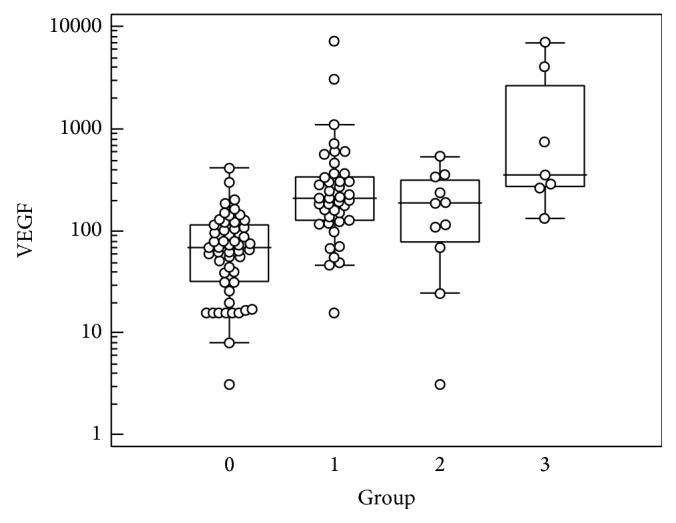
Vascular endothelial growth factor levels for each group. Group 1: proliferative diabetic retinopathy (PDR) patients with no need for repeated surgical intervention; group 2: PDR patients with repeated vitreous bleeding, which is a less serious complication of PDR; group 3: PDR patients with refractory neovascular glaucoma, which is a serious complication of PDR; group 0: control group.

**Figure 2:**
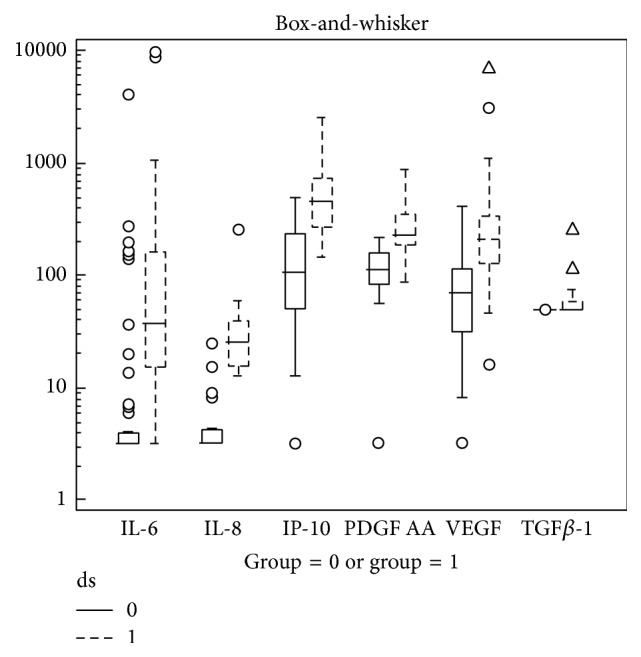
Levels of biomarkers for which significant differences between the levels in control group 0 and those in proliferative diabetic retinopathy group 1 were found. IL: interleukin; IP-10: *γ*-interferon inducible protein 10; PDGF AA: platelet-derived growth factor AA; VEGF: vascular endothelial growth factor; TGF*β*-1: transforming growth factor beta 1.

The median, lower, and upper quartile values for all of the markers within each group are listed in Table 1. When comparing the groups, significantly higher levels of IL-6, IL-8, IP-10, PDGF AA, and VEGF were found among PDR patients compared with patients in the control group. The concentrations of TGF*β*-1 were higher in PDR patients compared with the control group. Patients in group 3 (those with neovascular glaucoma that was refractory to treatment) had higher levels of IL-6, TGF*β*-1, and VEGF compared with patients in PDR group 1 and PDR group 2 (the nonneovascular glaucoma groups). The differences in concentrations were all of borderline significance (see Tables 1 and 2). No significant differences in marker levels were found between PDR group 1 (with no complications) and PDR group 2 (with repeated vitreous bleeding). No differences between groups were found in the levels of BDNF, CNTF, EGF, and MCP-1. See Table 2 for the results of the group comparisons. Boxplots of the VEGF concentrations in each of the groups are provided in Figure 1, and boxplots of the markers for which levels in control eyes differed significantly from levels in the eyes of patients in PDR group 1 are shown in Figure 2. Because the vast majority of patients had intraocular fluid concentrations of fractalkine, PDGFAB/BB, IL-10, IFN-*γ*, TNF-*α*, FGF-2, and RANTES that were below the detection limit of the panels that were used, the results from assays for these markers are not presented. Although the concentration of TGF*β*-1 was below the detection limit in the control group, TGF*β*-1 levels in some of the PDR patients were measureable; thus, the results are presented.

## 4. Discussion

Biomarkers in disease detection and management have become important tools in modern clinical medicine, and their application to retinal disease should be no exception. Because multiplex analysis based on xMAP technology allows for the analysis of tens of analytes in a small sample volume (10–50 *μ*L), this is a potent technology for introducing laboratory medicine into ophthalmology.

In this study, we have confirmed that the patients with PDR have higher intraocular concentrations of proangiogenic, antiangiogenic, and inflammatory cytokines compared with nonPDR patients. Intraocular levels of IL-6, IL-8, IP-10, PDGF AA, TGF*β*-1, and VEGF were increased in patients with PDR. Today, many studies compare the intraocular concentrations of various cytokines in PDR patients versus patients who do not have PDR. Maier et al. found that mean cytokine levels of IP-10, MCP-1, and VEGF in the vitreous humour were significantly higher compared to those of normal controls [8]. Murugeswari et al. documented that levels of IL-6, IL-8, MCP-1, and VEGF in the vitreous were significantly higher in PDR patients compared with levels in macular hole patients. Conversely, the vitreous level of PEDF was significantly reduced in patients with PDR [9]. Yoshimura et al. performed a comprehensive analysis of mediators in the vitreous fluids in PDR patients and in patients with other ocular diseases, and they found elevated levels of VEGF, MCP-1, IL-8, and IL-6 compared with control patients [10]. We found similar results in this study, but we have not demonstrated that the concentration of MCP-1 increases in patients with PDR. However, we have shown that higher intraocular concentrations of PDGF AA and nonmeasurable values of PDGF AB/BB can be seen in PDR patients. Contrary to our result, Freyberger et al. published results showing that PDGF AB levels are elevated in patients with PDR [11].

In a clinical environment, it is essential to further stratify the PDR patients; however, only a few studies that compare the levels of biomarkers in PDR patients with differing disease severities exist. Funatsu et al. divided PDR patients into subgroups based on disease progression and regression. The vitreous levels of VEGF and IL-6 were significantly higher in the eyes of patients in the progression group than they were in eyes with PDR regression. Multivariate logistic regression analysis showed that higher vitreous levels of VEGF were associated with the progression of PDR following vitreous surgery. A high vitreous level of VEGF was identified as a significant risk factor in determining the outcome of vitreous surgery in patients with PDR [12]. Freyberger et al. studied 23 patients with PDR, four of whom had rubeosis iridis, which is an indicator of very high vasoproliferative activity. Significantly elevated concentrations of PDGF AB were found among individuals with PDR; even higher levels were found in conjunction with rubeosis iridis [11]. In our study, patients with neovascular glaucoma that was refractory to treatment showed higher levels of IL-6, TGF*β*-1, and VEGF than other PDR patients, which implies that the levels of these three factors are correlated with the severity of PDR. No differences in biomarker levels were found between patients who belonged to group 2 (those with repeated vitreous bleeding) and those who belonged to group 1 (those who had no complications).

The novel multiplex technology that we proposed not only saves time, labor, and costs of immunoanalysis, but it also rapidly reduces the sample volume requirements compared to a traditional immunoanalysis method (single ELISA) while allowing the full comparability of all studied parameters. The last two points are critical when entering laboratory measurements into the diagnostic and risk assessment process in ophthalmology. One limitation of xMAP technology could be that it is limited in its ability to detect some factors. In the present study, we were not able to detect fractalkine, PDGF AB/BB, IL-10, IFN-*γ*, TNF-*α*, FGF-2, and RANTES in the aqueous humour. Similarly, Yoshimura et al. found that the intraocular concentrations of IL-1*β*, IL-2, IL-4, IL-5, IL-10, IL-17, IFN-*γ*, TNF-*α*, eotaxin, MIP-1*α*, RANTES, EGF, and FGF-2 were lower than the detection level [10].

We have chosen three works as examples of studies that have shown the potency of xMAP technology in ophthalmology. Curnow et al. measured a panel of cytokines in the aqueous humour, and from the spectra of cytokines that they studied, they used random forest analysis to show that only IL-6, IL-8, MCP-1, IL-13, IL-2, and TNF-*α* are required to distinguish between noninflammatory control and idiopathic uveitis with 100% classification accuracy [13]. Funding et al. used xMAP technology to simultaneously quantify and compare the concentrations of 17 immune mediators in aqueous humour samples from patients with corneal rejection and patients with a noninflammatory condition in the anterior chamber. Their results underscore both the complex immunological interactions of the rejection process and the need for multiplex laboratory measurements based on small sample volumes [14]. Rusnak et al. measured the levels of 12 cytokines in the aqueous humour in 27 eyes that were undergoing vitrectomies for retinal detachment with various degrees of severity of proliferative vitreoretinopathy. According to this study MCP-1 and VEGF may participate in pathogenesis of retinal detachment and proliferative vitreoretinopathy [15].

Sohn et al. have shown that multiplex measurements of cytokine and growth factor concentrations also enable treatment monitoring. After intravitreal injections of 2 antiangiogenic drugs (triamcinolone and bevacizumab), the clinical effects and differences in biomarker levels in the aqueous humour were monitored. A more effective treatment modality was linked to decreases in the concentrations of IL-6, IP-10, MCP-1, PDGF AA, and VEGF compared with those resulting from a less effective treatment; the latter treatment was only connected with a decrease in the concentration of VEGF [16].

The Sohn et al. study [16], in conjunction with our findings in patients with neovascular glaucoma refractory to treatment, shows that biomarkers have a strong potential for use in patient stratification and in determining personalized medical needs. Tailored treatments are necessary due to the introduction and costs of novel treatment. The vast majority of novel types of therapy are based on the inhibition of VEGF. A number of anti-VEGF agents have been introduced into clinical use and are widely used for the treatment of many ocular diseases, but the widespread use of these agents raises new questions. It has been proposed that anti-VEGF agents may have negative effects on retinal cells. Animal studies have shown that systemic neutralization of VEGF with soluble VEGF receptors results in a reduction of the thicknesses of both the inner and the outer nuclear layers in adult mouse retinas. These results indicate that endogenous VEGF plays an important role in the maintenance and function of neuronal cells in the adult retina and suggest that anti-VEGF therapies should be administered with caution [17]. Because of the risks associated with using anti-VEGF therapies, it is absolutely necessary to select patients who can benefit from anti-VEGF treatment despite the risk of adverse effects. In our study, we have shown that certain complications, such as neovascular glaucoma that is refractory to conventional treatment, are correlated with high concentrations of certain biomarkers; in the future, we can use these to justify more aggressive therapies. Our findings suggest that patients could be selected for repeat intravitreal injections of VEGF inhibitors, corticosteroids, more aggressive panretinal laser photocoagulation, cyclocryodestruction, or cyclophotodestruction on the basis of biomarker concentrations. Another study that shows that measuring protein concentrations in the aqueous humour has potential future benefits for treatment monitoring was conducted by Campochiaro et al.; they measured concentrations of VEGF, IL-6, IL-1 beta, tumor necrosis factor, and ranibizumab [18].

Another problem in patients who have been treated with anti-VEGF therapies is determining the concentration of VEGF. The determination of the VEGF concentration is influenced by treatment with anti-VEGF inhibitors via direct interaction in the immunoanalysis, which we verified in our laboratory (data not presented). The interaction requires an adjustment to the approach to determining the intraocular concentrations of VEGF in these patients; the half-life of anti-VEGF therapies in the eye was established as being 9.8 days [19]. Only patients who had never received anti-VEGF treatment or, in advanced cases, had received their most recent administrations of anti-VEGF therapy more than two months prior to aqueous humour sampling for this study were included. With the expansion of anti-VEGF therapy, it is clear that multifactor monitoring, that is, introducing other biomarkers in addition to VEGF, is important. Both our study and others show that there are several possible candidate biomarkers. As more PDR biomarkers are identified, a panel of them has the potential to be effective for identifying high-risk individuals, monitoring disease progression, and evaluating the efficacy of therapeutic interventions.

In conclusion, the results of our study suggest that the concentrations of IL-6, TGF*β*-1, and VEGF correlate with the severity of PDR. In future, assessment of PDR biomarkers in intraocular fluid could be effective method for treatment monitoring and early detection of PDR progression.

## Figures and Tables

**Table 1:** Descriptive statistics. Median values and 5th and 95th percentile values in pg/mL for all markers and groups are shown. Proliferative diabetic retinopathy (PDR) patients were divided into groups. Group 1: PDR patients with no need for repeated surgical intervention; group 2: PDR patients with repeated vitreous bleeding, which is a less serious complication of PDR; group 3: PDR patients with refractory neovascular glaucoma, which is a serious complication of PDR; group 0: control group.

	Groups							
	0		1		2		3	
	Median	5–95 P	Median	5–95 P	Median	5–95 P	Median	5–95 P
BDNF	12.00	12.00–59.67	12.00	12.00–49.37	12.00	12.00–103.23	30.00	12.00–81.43
CNTF	122.00	122.00–2667.92	393.19	122.00–1787.45	276.19	122.00–573.63	388.29	197.96–578.61
EGF	24.88	13.893–115.99	36.22	14.02–133.31	96.47	4.23–120.42	37.48	3.95–114.20
IL-6	3.20	3.200–196.312	37.19	3.992–4577.38	25.22	3.37–299.13	384.74	22.63–9982.69
IL-8	3.20	3.20–15.49	25.28	13.21–184.62	27.96	13.84–53.67	33.76	20.04–96.37
IP-10	105.92	12.93–307.68	460.68	159.49–2237.28	365.35	150.90–758.61	874.06	463.94–1698.36
MCP-1	962.59	3.20–2931.63	2772.21	353.18–3249.45	2336.77	2020.39–2653.15	661.23	3.20–2881.94
PDGFAA	111.26	5.85–212.85	227.96	104.57–756.34	208.36	151.58–344.62	192.66	79.91–316.61
TGF*β*-1	49.00	49.00-49.00	49.00	49.00–220.41	49.00	49.00–84.71	414.40	119.85–955.58
VEGF	69.85	16.00–200.63	211.62	48.10–1990.98	187.96	4.28–523.25	352.82	132.84–7052.89

BDNF: brain-derived neurotrophic factor; CNTF: ciliary neurotrophic factor; EGF: epidermal growth factor; IL: interleukin; IP-10: *γ*-interferon inducible protein 10; MCP-1: monocyte chemoattractant protein 1; PDGF AA: platelet-derived growth factor AA; TGF*β*-1: transforming growth factor beta 1; VEGF: vascular endothelial growth factor.

P: percentile.

**Table 2:** Comparison of biomarker levels between groups. *P* values are listed.

	Kruskal-Wallis	Mann-Whitney *U* 0 × 1	Mann-Whitney *U* 1 × 2	Mann-Whitney *U* 2 × 3	Mann-Whitney *U* 1 × 3
BDNF	NS	NS	NS	NS	NS
CNTF	NS	NS	NS	NS	NS
EGF	NS	NS	NS	NS	NS
IL-6	<0.0001	<0.0001	NS	0.0088	0.0096
IL-8	<0.0001	<0.0001	NS	NS	NS
IP-10	<0.0001	<0.0001	NS	NS	NS
MCP-1	NS	NS	NS	NS	NS
PDGFAA	<0.0001	<0.0001	NS	NS	NS
TGF*β*-1	<0.0001	0.0027	NS	0.0037	0.0017
VEGF	<0.0001	<0.0001	NS	0.0265	0.0454

BDNF: brain-derived neurotrophic factor; CNTF: ciliary neurotrophic factor; EGF: epidermal growth factor; IL: interleukin; IP-10: *γ*-interferon inducible protein 10; MCP1: monocyte chemoattractant protein 1; PDGF AA: platelet-derived growth factor AA; TGF*β*-1: transforming growth factor beta 1; VEGF: vascular endothelial growth factor.

NS: nonsignificant.
